# Supplementary figures and images for: Interference of Celastrol with Cell Wall Synthesis and Biofilm Formation in Staphylococcus epidermidis
Source: Antibiotics (Basel). 2025 Jan 3;14(1):26. doi: 10.3390/antibiotics14010026 (PMC11759760; doi:10.3390/antibiotics14010026)

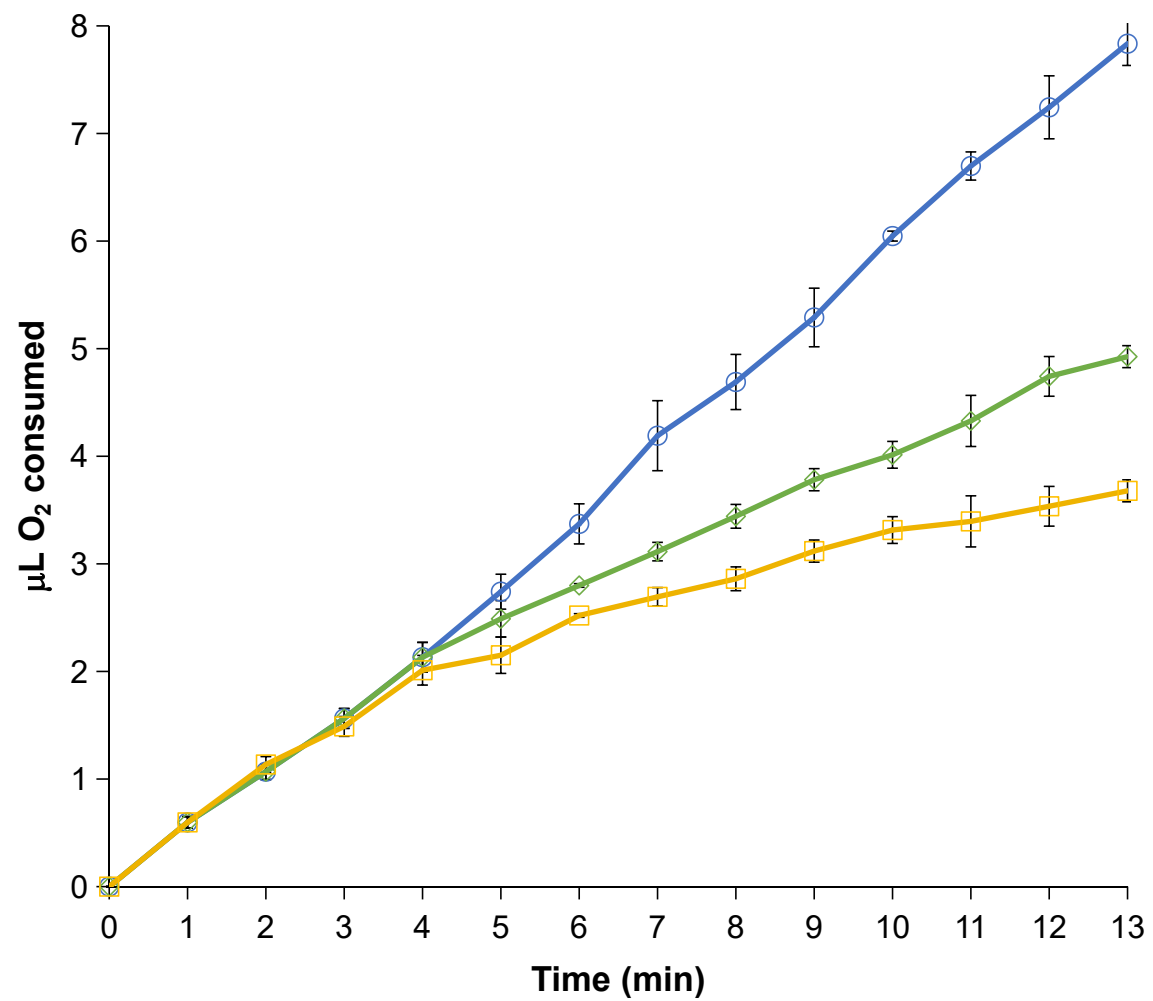

Control

Celastrol

NaCN 2M

Supplement: Supplementary file 1 [file antibiotics-14-00026-s001.zip › antibiotics-3394078-supplementary.pdf]
